# Low-glycemic index functional flour modulates gut microbiota in association with anti-obesity effects in mice

**DOI:** 10.3389/fnut.2026.1910403

**Published:** 2026-10-13

**Authors:** Aijun Tong, Ying Zheng, Wenfang Cheng, Junlin Wu, Yueting Zheng, Simin Liu, Xiaoyan Liu, Chao Zhao, Bin Liu

**Affiliations:** 1 College of Tea and Food Science, Wuyi University, Wuyishan, China; 2 College of Food Science, Fujian Agriculture and Forestry University, Fuzhou, China; 3 College of Marine Sciences, Fujian Agriculture and Forestry University, Fuzhou, China; 4 School of Food and Health, Beijing Technology and Business University, Beijing, China

**Keywords:** gut microbiota, *Laminaria japonica*, *Lentinula edodes*, lipid metabolism, low-glycemic index flour

## Abstract

**Background:**

Obesity has become a prevalent global chronic disease, and dietary intervention is a safe and effective strategy for obesity prevention and management. *Laminaria japonica* and *Lentinula edodes* are widely consumed edible resources in China, while the lipid-lowering efficacy of functional noodles supplemented with their dietary fibers remains unclear. Moreover, the potential synergistic effects of combining these fibers with other functional plant ingredients have not been systematically explored.

**Methods:**

This study developed three low-glycemic index (low-GI) functional flour formulations (R1: basic formula; R2: basic formula supplemented with *L. japonica* and *L. edodes* dietary fibers; R3: R2 fortified with additional functional starches) based on nutritional balance principles. The *in vitro* nutritional properties, digestibility, cholesterol and bile salt binding capacities, as well as α-glucosidase and pancreatic lipase inhibitory activities were evaluated. Furthermore, a high-fat diet-induced obese ICR mouse model was established to evaluate the *in vivo* regulatory effects of the three formulations via a 6-week dietary intervention.

**Results:**

All three formulations exhibited GI values below 55 and showed significantly lower starch hydrolysis rates compared with wheat flour (WF), along with enhanced cholesterol adsorption capacity and bile salt-binding capacities, as well as stronger α-glucosidase and pancreatic lipase inhibitory activities. In the animal experiments, all three formulations significantly reduced mouse body weight, alleviated liver damage and hepatocellular pathology, reduced epididymal adipocyte size, and attenuated cecal tissue inflammation, with R3 showing the most pronounced effects. Notably, Formula 3 (R3) treatment also significantly modulated gut microbiota composition, as evidenced by increased beneficial bacteria, a reduced Firmicutes/Bacteroidetes (F/B) ratio, decreased potentially pathogenic taxa, and elevated fecal short-chain fatty acids (acetic, propionic, butyric, and isobutyric acids) compared with the model group. Correlation analyses revealed significant associations between specific microbial genera and metabolic parameters.

**Conclusion:**

These findings suggest that the low-GI functional flours, particularly R3, improve metabolic health in obese mice, accompanied by modulation of gut microbiota and short-chain fatty acids (SCFAs) production. However, given the correlational nature of the current data, causal relationships need to be confirmed through further mechanistic studies, such as fecal microbiota transplantation. Overall, R3 represents a promising dietary intervention for obesity management.

## 1 Introduction

Obesity is recognized as a chronic and relapsing disease. It affects over 1 billion individuals worldwide, and its prevalence continues to rise steadily (1). The condition poses substantial public health challenges through its association with numerous comorbidities, including increased incidence of non-alcoholic fatty liver disease, type 2 diabetes, cardiovascular diseases, hepatic disorders, and various malignancies. The pathological state often involves metabolic disturbances, including dyslipidemia, insulin resistance, and gut microbial dysbiosis (2). Dietary patterns are one of the factors that affect this complex and multifactorial disease state, exhibiting characteristic features of chronicity, tendency for relapse, and progressive nature (3). However, pharmacotherapy has limitations, such as the requirement for extended treatment duration and potential adverse effects ranging from emotional disorders to cardiovascular and cerebrovascular diseases (4). In clinical practice, dietary modification is increasingly recognized as a fundamental strategy for obesity prevention and management, given its superior safety profile and cost-effectiveness (5).

The gut functions as a critical immune organ that hosts a dynamic and highly diverse microbial ecosystem. These microbial communities play a pivotal role in regulating both physiological and pathological processes through their influence on metabolic processes and gut immune responses (6). Comparative analyses have demonstrated that obese individuals exhibit distinct gut microbiota alterations, characterized by decreased Bacteroidetes and increased Firmicutes populations. The elevated F/B ratio exacerbates metabolic dysregulation, fostering systemic inflammation and metabolic disorders (7). Studies indicate that gut microbiota metabolize dietary carbohydrates to enhance SCFAs production, thereby supplying additional energy substrates that contribute to lipid deposition and glucose storage (8). SCFAs such as acetic acid, propionic acid, butyric acid, valeric acid, isobutyric acid, and isovaleric acid serve as key mediators linking diet, gut microbiota, metabolism, and immunity. They play crucial roles in regulating processes including cell apoptosis, inflammation, and lipid metabolism (9). These bioactive compounds serve as important mediators in host microbiome crosstalk, influencing various physiological processes and playing established roles in metabolic disorders including obesity, diabetes and cardiovascular diseases (10). Accumulating research highlights the potential of dietary fiber (DF) to modulate gut microbiota composition, enhance antioxidant defenses, improve insulin sensitivity, and regulate key enzymes involved in glucose and lipid metabolism, thereby exerting beneficial hypoglycemic and hypolipidemic effects (11, 12).

Low GI dietary patterns contribute to slowing carbohydrate absorption kinetics, prolonged satiety signals, reduce energy intake, stabilizing postprandial glycemia, and promoting weight management (13). Mechanistically, the anti-obesity mechanisms of low-GI dietary interventions operate through four primary pathways: suppression of postprandial glycemic excursions and insulin response stabilization, enhancement of lipid oxidation capacity accompanied by a reduction in circulating free fatty acids, potential modulation of gut microbiota derived SCFAs production, and stimulation of satiety-related peptide secretion. These mechanisms collectively promote improved lipid homeostasis and body weight regulation (14). From a nutritional perspective, foods are categorized as high-GI (GI > 75) or low-GI (GI ≤ 55), with the latter typically exhibiting favorable nutritional profiles including lower energy density and higher DF content. Soluble DF plays a pivotal role in reducing the GI of flour products by hindering the interaction between digestive enzymes such as amylase, and starch, thereby curbing enzymatic activity (15). Resistant starch (RS), a healthy DF, directly reduces postprandial blood glucose levels and serum cholesterol levels, it also improves the glycemic response and has potential applications in high-quality food products (16).

Currently, the exploration of novel ingredients for partial replacement of WF, and the development of low-GI foods rich in DF, have become key research focuses. *L. japonica* and *L. edodes* are rich in DF and various bioactive compounds (17, 18). Previous studies have demonstrated that sulfated fucoidan derived from *L. japonica* modulates lipid metabolism in mice via the bile acid-mediated intestinal FXR-FGF19-CYP7A1/CYP8B1 pathway (19). Soluble DF extracted from *L. edodes* has been shown to reduce systemic levels of triglycerides (TG), low-density lipoprotein cholesterol (LDL-C), free fatty acids (FFA). These changes collectively contribute to the amelioration of high-fat diet-induced liver injury, oxidative stress, and disorders of lipid metabolism (20). The unique nutritional compositions and bioactive properties position *L. japonica* and *L. edodes* as promising candidates for the development of novel, health-promoting food products targeting weight management and metabolic health. Similarly, tartary buckwheat, kudzu root and konjac are three medicinal and edible plants that have attracted considerable research interest due to their potential in managing metabolic disorders. Tartary buckwheat is rich in bioactive components, including RS, DF, protein, and especially phenolic substances, which contribute to its protective effects against hypertension, obesity, and cardiovascular diseases (21). Kudzu root is rich in isoflavones, polysaccharides, and RS, which have been reported to exert insulin-sensitizing, anti-inflammatory, antioxidant, and gut microbiota-modulating activities (22–24). Konjac is characterized by its high content of glucomannan, which is a soluble dietary fiber, along with flavonoids and phenolic compounds. These components collectively contribute to weight management, glycemic stabilization, and lipid profile improvement, as well as beneficial effects on intestinal health, inflammation, and immune regulation (25).

*Laminaria japonica*, *L. edodes*, tartary buckwheat, kudzu root and konjac are all rich in flavonoids, polyphenols and bioactive polysaccharides, which have been proven to alleviate glycolipid disorders. Each raw material exhibits distinct hypoglycemic and anti-obesity bioactivities, yet their synergistic combination for low-GI functional flour remains underexplored. Accordingly, this study compounded the five raw materials to prepare a novel low-GI flour, hypothesizing that their abundant shared bioactive components would jointly regulate glucose and lipid metabolism. We adopted dietary fiber from *L. japonica* and *L. edodes* as the base material, characterized its nutritional and *in vitro* functional properties, and verified it’s *in vivo* effects on glycolipid metabolism, gut microbiota and SCFAs using obese mice, to provide theoretical support for anti-obesity functional food development.

## 2 Materials and methods

### 2.1 Raw materials and preparation

WF, tartary buckwheat flour, oat flour, barley flour, rye flour, quinoa, *L. japonica, L. edodes*, and other raw materials were obtained from a local supermarket. *L. japonica and L. edodes* were dried at 40 °C for 24 h, pulverized, and sieved through 40–200 mesh. The dried powders were extracted with distilled water at a solid-to-liquid ratio of 1:40–80 using a compound enzyme (pectinase:cellulase = 1:2, 0.5–3% of the raw material) at 50–90 °C and pH 5–8 for 2–6 h. After enzyme inactivation at 100 °C for 10 min, the mixture was centrifuged (4,000 rpm, 25 min). The supernatant was concentrated and dried to obtain the aqueous extract. The residue was further hydrolyzed with 0.5–3% complex protease at a solid-to-liquid ratio of 1:40–80 and pH 5–8 at 50–90 °C for 2–6 h, followed by centrifugation (4,000 rpm, 25 min). The supernatant was concentrated and dried to obtain the peptide fraction. The remaining residue was extracted with 0.5–3% sodium carbonate solution at a solid-to-liquid ratio of 1:40–80 and 55–95 °C for 2–6 h. After centrifugation (4,000 rpm, 25 min), the supernatant was precipitated with four volumes of anhydrous ethanol for 8 h and dried to obtain soluble dietary fiber, and the residual precipitate was dried to obtain insoluble dietary fiber. All fractions were lyophilized and stored at −20 °C until further use (26).

### 2.2 Formulation of flour blends

Based on the principles of balanced dietary nutrition and the GI, we developed three formulated flours targeted at blood glucose and lipid management. The detailed compositions are presented in Table 1. The three formulations were designed using a stepwise optimization strategy: R1 served as the base formulation; R2 was formulated by incorporating DFderived from *L. japonica* and *L. edodes* into the base formulation; and R3 was further supplemented with additional functional ingredients, including yam, Poria cocos, kudzu root, and white kidney bean powders, to achieve enhanced nutritional and functional properties. All three flour formulations were prepared in a single batch to ensure consistency throughout the animal study, following the procedures described in Section 2.3. Each flour was stored under vacuum-sealed conditions at 4 °C prior to diet preparation.

**Table 1:** Composition of raw materials in the formula.

Formula ID	Main formula
R1	70% WF, 10% tartary buckwheat flour, 5%tremella fuciformis flour, 7% konjac flour, and 8% soybean flour
R2	40% WF, 10% tartary buckwheat flour, 15% *L. japonica* DF, 15% *L. edodes* DF, 5% tremella fuciformis flour, 7% konjac flour, and 8% soybean flour
R3	20% WF, 10% tartary buckwheat flour, 15% *L. japonica* DF, 15% *L. edodes* DF, 5% soybean flour, 7% tremella fuciformis powder, 5% yam powder, 5% poria cocos powder, 5% kudzu root powder, 8% konjac flour, 5% white kidney bean powder

### 2.3 Basic components and *in vitro* activity of formula powder

#### 2.3.1 Determination of basic components of formula flour

The formulated flour mainly comprises starch, DF, protein, fat and other minor constituents. Moisture, protein and fat contents were determined following AOAC 930.15 (27), GB 5009.5–2016 (28) and GB 5009.6–2016 (29) respectively. Total DF content was quantified in accordance with GB 5009.88–2023 (30). Total flavonoid content was assayed by the aluminum chloride colorimetric method using rutin as the standard, with absorbance recorded at 510 nm (31). Total polyphenol content was determined via the Folin–Ciocalteu method (gallic acid as standard), and absorbance was measured at 765 nm (32). Total carbohydrate content was calculated by difference:

Total carbohydrate (%) = 100 − (moisture + protein + fat + ash) %

#### 2.3.2 Digestion characteristics and eGI

The *in vitro* digestion assay, modified from a previously reported protocol (33), was conducted individually on each cooked and homogenized flour sample. Briefly, each sample (0.2 g) was incubated with 10 mL sodium acetate buffer (0.2 mol/L, pH 5.2) at 37 °C for 5 min under stirring at 170 rpm, followed by the addition of enzyme solution containing α-amylase (290 U/mL) and amyloglucosidase (15 U/mL). Aliquots were collected at predetermined time intervals, and glucose concentrations were quantified using the 3,5-dinitrosalicylic acid (DNS) method. White bread was adopted as the reference sample to calculate the hydrolysis index (HI). Starch hydrolysis data were fitted to a first-order kinetic equation, and the HI and estimated glycemic index (eGI) were calculated following the established procedure as described below (34):


$$\mathrm{Ct}=C\infty (1-e^{-\mathrm{kt}})$$

$$\mathrm{HI}\;(\%)=({\mathrm{AUC}}_{\text{sample}}/{\mathrm{AUC}}_{\text{white bread}})\times 100$$

eGI=0.862×HI+8.1981

Where Ct (%) is the starch hydrolysis at time t (min), C∞ is the final hydrolysis rate, and k (min^−1^) is the digestion rate constant.

#### 2.3.3 Cholesterol and sodium cholate adsorption capacities

The cholesterol and sodium cholate adsorption capacities were determined using a previously reported procedure (35). Briefly, each flour sample (2.0 g) was mixed with 50 mL diluted egg yolk emulsion (liquid-to-solid ratio = 25 mL/g), and the mixture was adjusted to pH 7.0, followed by incubation at 37 °C for 2 h under shaking. After centrifugation at 5,000 rpm for 15 min, the cholesterol concentration in the supernatant was quantified using the o-phthalaldehyde method. The adsorption capacity was calculated as follows:


$$(W_{1}-W_{2})/m$$

Where W₁ is the initial cholesterol content, W₂ is the supernatant cholesterol content, and m is the sample weight (g).

Sodium cholate adsorption was assessed using a simulated gastrointestinal digestion model. Each flour sample (8 g/100 mL) was incubated with simulated gastric fluid (pH 3.0, 2,000 U/mL pepsin) at 37 °C for 4 h with shaking (150 rpm), followed by heat inactivation. After adjusting to pH 7.0, the sample was incubated with simulated intestinal fluid (pH 7.0, 100 U/mL trypsin, 4 USP/mL pancreatin, 10 mmol/L bile salts) at 37 °C for 4 h. After centrifugation (8,000 rpm, 10 min), the supernatant (1 mL) was mixed with sodium cholate solution (1 mmol/L, 4 mL) and incubated at 37 °C for 1 h. Residual bile salt content was measured at 387 nm. Adsorption rate was calculated as follow:


(L–H)/L×100

Where L is total sodium cholate added and H is the supernatant content.

All measurements were performed in triplicate, with standard curves established for cholesterol (*R*^2^ = 0.9901) and sodium cholate (*R*^2^ = 0.9922).

#### 2.3.4 α-Glucosidase and pancreatic lipase inhibition activity

The α-glucosidase inhibitory activity was determined with slight modifications based on the method by Manzoor et al. (36) using p-nitrophenyl-α-D-glucopyranoside as the substrate. Reactions and absorbance measurements were performed in a 96-well microplate using a SpectraMax i3x microplate reader (Molecular Devices), and inhibition rates were calculated relative to control wells. All measurements were performed in triplicate. Inhibitory activity against porcine pancreatic lipase was evaluated with adjustments based on Manzoor’s methods. The substrate solution was prepared by dissolving 0.08 g of 4-nitrophenyl laurate in 100 mL of sodium acetate buffer (5 mM, pH 5.0) containing 1% Triton X-100. Porcine pancreatic lipase solution (5 mg/mL) was prepared in ultrapure water and centrifuged (12,000 rpm, 10 min), and the supernatant was collected. The sample supernatant, pancreatic lipase solution, and buffer were mixed and incubated, followed by the addition of substrate. Absorbance was measured at 405 nm. Inhibition rates were calculated relative to control wells. All measurements were performed in triplicate.

### 2.4 The effect of formula flour on glucose and lipid metabolism in mice

#### 2.4.1 Experimental animals

Four-week-old healthy male ICR mice were purchased from Fuzhou Wu’s Animal Center (Fuzhou, China). The animals were housed under standardized laboratory conditions: temperature of 25 ± 5 °C, relative humidity of 50 ± 10%, and a 12 h light/dark cycle. After a 10-day acclimatization period, the mice were randomly divided into two groups based on body weight: a normal group (normal, *n* = 12) fed a standard maintenance diet (HFK 1022, Beijing Huafukang Biotechnology Co., Ltd., China, 3.42 kcal/g, consisting of 22.47% protein, 12.11% fat, and 65.42% carbohydrates), and an obesity induction group (*n* = 58) fed a high-calorie diet composed of 15% sucrose, 15% lard, 1% cholesterol, 10% egg yolk powder, 0.1% sodium cholate, and 58.9% basal maintenance feed (w/w). After 2 weeks of high-calorie diet feeding, 48 mice that met the obesity criterion (body weight ≥ 20% above the mean weight of the normal group) were randomly assigned to four groups (*n* = 12 per group). All experimental procedures, including diet administration, body weight measurement, and sample collection, were performed by investigators blinded to the group allocation. During the 6-week intervention period, the high-calorie diet was discontinued, and all groups received experimental diets containing 70% of the respective flour and 30% basal nutritional components (w/w). The normal and model groups received 70% normal WF, while the R1, R2, and R3 groups received 70% formula 1, 2, and 3 flour, respectively. The basal components were identical across all diets. The normal group differed from the model group only in that it did not undergo the 2-week high-calorie induction. The sample size (*n* = 12 per group) was determined based on previous studies to ensure sufficient statistical power for detecting significant differences in metabolic parameters (37).

#### 2.4.2 *In vivo* GI determination

Prior to the obesity induction described in Section 2.4.1, all acclimatized mice were subjected to *in vivo* GI measurements. After a 12 h fast, basal blood glucose levels were measured. Mice were then randomized into five groups and orally administered glucose, WF, or one of the three formulated flours (R1-R3) at 0.72 g/kg bw (equivalent to 50 g sample per 65 kg adult male, based on body surface area scaling) (38). Blood samples were collected from the tail tip at 0, 30, 60, 90, and 120 min post-gavage. Blood glucose response curves were plotted, and the area under the curve (AUC) was calculated using Origin 2021. GI values were calculated as:


$$\mathrm{GI}=({\mathrm{AUC}}_{\text{sample}}/{\mathrm{AUC}}_{\text{glucose}})\times 100$$

Following the test, mice were given a 3-day recovery period before proceeding with the obesity induction in Section 2.4.1.

#### 2.4.3 Energy intake calculation

The energy density (ED) of each flour formulation was calculated using the Atwater general factor system, as recommended by the FAO (39). The following conversion factors were applied:


$$\begin{array}{l} {\mathrm{ED}}_{R}(\text{kcal}/100\;g)=\text{Protein}\;(\%)\times 4+\mathrm{Fat}\;(\%)\times 9\\ +\text{Carbohydrate}\;(\%)\times 4+\mathrm{DF}\;(\%)\times 2 \end{array}$$

$$\text{Complete diet}\;\mathrm{ED}=(0.7\times {\mathrm{ED}}_{R})+(0.3\times {\mathrm{ED}}_{\mathrm{WF}})$$

Daily food intake (g/mouse/day) was calculated by dividing the weekly food intake (g/mouse/week) by 7. Daily energy intake (kcal/mouse/day) was then calculated as: Daily energy intake = Daily food intake × Diet energy density.

#### 2.4.4 Blood glucose changes and oral glucose tolerance assays

Mice were subjected to a 12-h fasting period (with ad libitum access to water) at 7-day intervals, blood samples were collected from the tail vein of the mice. Fasting blood glucose (FBG) levels were measured using a glucometer (HGM-112, Omron Health Medical (China) Co., Ltd.) with compatible test strips to evaluate differences across experimental groups. Following a 6-week dietary intervention, mice were fasted for 12 h (water permitted) prior to the oral glucose tolerance test (OGTT) measurement (baseline, 0 h). Subsequently, an oral glucose solution (2 g/kg body weight) was administered. Blood glucose levels were assessed at 0.5, 1, and 2 h post-administration. Glucose tolerance curves were generated by plotting the four time-point measurements (0 h, 0.5 h, 1 h, and 2 h), and the AUC-OGTT was calculated for each group to determine glucose tolerance capacity.


$$\begin{array}{l} \mathrm{AUC}-\text{OGTT}=0.25\times (G0\;h+G0.5\;h)+0.25\times (G0.5\;h+G1\;h)\\ +0.5\times (G1\;h+G2\;h) \end{array}$$

Where G0 h, G0.5 h, G1 h, and G2 h represent blood glucose concentrations at baseline (0 h), 0.5 h, 1 h, and 2 h after glucose administration, respectively.

#### 2.4.5 Serum biochemical assays

Following a 6-week dietary intervention, mice were intraperitoneally anesthetized with ketamine (100 mg/kg) and xylazine (10 mg/kg). Once deep anesthesia was confirmed, blood samples (0.5–1.0 mL) were collected via eyeball enucleation, a method adopted to acquire sufficient non-hemolyzed whole blood rapidly with minimal cumulative stress to experimental animals for subsequent multi-index analyses. Blood samples were collected, clotted at room temperature for 1.5 h, and centrifuged at 5000 rpm for 15 min at 4 °C to obtain serum. The serum was aliquoted and stored at −80 °C pending subsequent analysis. Serum levels of TG, total cholesterol (TC), LDL-C, high-density lipoprotein cholesterol (HDL-C) and FFA were quantified using commercially available assay kits (Jiancheng Institute of Biotechnology, Nanjing, China), following the manufacturer’s instructions. After blood collection, mice were euthanized by cervical dislocation. Epididymal and perirenal fat pads were dissected and weighed, and fat coefficients were calculated as:


Fatcoefficient(%)=(fatpadweight/body weight)×100

$${\mathrm{Lee}}^{’}s\;\text{index}=(g/\mathrm{mm})=\sqrt[{3}]{\begin{array}{l} \text{body weight}\times 1000/\\ \text{body length}) \end{array}}\;(g/\mathrm{mm})$$

Where body length was measured as the naso-anal distance.

#### 2.4.6 Histological examination

The liver, cecum, and epididymal adipose tissue of mice were fixed with 4% paraformaldehyde for 24 h. After dehydration and paraffin immersion, paraffin embedding, and sectioning, the tissue morphology changes were observed, photographed, and analyzed using an optical microscope. Quantitative analyses were performed by two blinded observers using ImageJ software. For each animal, three random fields of view were analyzed, and the mean value was used as an independent biological replicate (n = 3). Hepatic lipid droplet area (%) was quantified in ×400 fields (40). Adipocyte cross sectional area (μm^2^) and radius (μm) were measured from at least 15 intact cells per ×100 field (41). Colonic crypt depth was measured in 5 crypts per ×100 field (42). Scale bars on the original images were used for calibration (50 μm for ×400; 200 μm for ×100).

#### 2.4.7 Gut microbiota analysis

Cecal contents samples were collected from mice in normal, model, and R3 groups (n = 5) and stored at −80 °C. Total genomic DNA was extracted using the CTAB method. The V3 + V4 hypervariable region of the 16S rRNA gene was amplified using the primers 341F (5’-CCTAYGGGRBGCASCAG-3′) and 806R (5’-GGACTACNNGGGTATCTAAT-3′) using GeneAmp 9,700 PCR instrument (Applied Biosystems, United States). Amplicons were sequenced on the Illumina NovaSeq 6,000 platform (2 × 300 bp). Raw reads were quality-filtered using fastp (Phred score < 20), and chimeric sequences were identified and removed using UCHIME (v4.2), clustered into operational taxonomic units (OTUs) at 97% similarity using UPARSE (v7.1), and taxonomically assigned using the SILVA database (v138) with a confidence threshold of 0.7. Alpha diversity (Shannon, Simpson, and Chao1) was calculated using QIIME2 (v2023.5). Beta diversity was assessed using Bray-Curtis PCoA and PERMANOVA (Adonis, 999 permutations) in the vegan package (v2.6–4). Homogeneity of group dispersions was tested using PERMDISP (betadisper function, followed by permutest, 999 permutations). Pairwise comparisons were corrected for multiple testing using Benjamini-Hochberg false discovery rate (FDR). LEfSe (v1.0) was used to identify differential taxa (LDA > 2, *p* < 0.05).

#### 2.4.8 Analysis of SCFAs

Cecal contents (150 mg) were mixed with 1 mL of ultrapure water and vortexed thoroughly. After adding 0.5 mL of phosphoric acid (6 M), the mixture was vortexed for 2 min. SCFAs were extracted twice with 1 mL of diethyl ether, with centrifugation at 1,000 rpm for 10 min at 4 °C after each extraction. The combined supernatant was filtered through a 0.22 μm organic membrane prior to GC–MS analysis. Standard curves for acetic acid, propionic acid, butyric acid, isobutyric acid, valeric acid, and isovaleric acid were each prepared using serial dilutions of the respective authentic standards at concentrations ranging from 10 to 5,245 μg/mL. The concentration of SCFAs in each sample was calculated based on peak area. GC–MS analysis was performed on an Agilent 8,860-5977B system equipped with an HP-INNOWAX capillary column (30 m × 0.32 mm × 0.25 μm, Agilent). The injection volume was 0.1 μL with a split ratio of 10:1. The injector and ion source temperatures were set at 240 °C and 230 °C, respectively. High-purity helium was used as the carrier gas at a flow rate of 1 mL/min. The oven temperature program was as follows: initial temperature 100 °C held for 5 min, then increased to 180 °C at 4 °C/min and held for 2 min.

### 2.5 Statistical analysis

All data are expressed as mean±standard deviation (SD). Statistical analyses were performed using SPSS 25.0 (IBM Corp., Armonk, NY, United States). normality was assessed using the Shapiro–Wilk test, and homogeneity of variances was assessed using Levene’s test. For comparisons among multiple groups, one-way analysis of variance was performed, followed by Duncan’s multiple range test for post-hoc comparisons. Student’s t-test was used for comparisons between two groups where applicable. Statistical significance was set at *p* < 0.05 and *p* < 0.01. Data visualization was conducted using Origin 2021 software (OriginLab Corporation, Northampton, MA, United States).

## 3 Results and discussion

### 3.1 Characterization of formula flour

Compared to ordinary WF, the three formulated flours (R1, R2, and R3) showed significantly enhanced nutritional profiles. Their protein content increased to 146.67, 146.67, and 138.3% of the WF value, respectively (*p* < 0.01), while carbohydrate content decreased to 87.07, 86.93, and 89.2%, respectively (*p* < 0.05). Additionally, the levels of key functional constituents, including dietary fiber (DF), polyphenols, and flavonoids, were all significantly elevated (*p* < 0.01). The content of DF was 2.2, 3.0, and 3.6 times that of WF, total polyphenol content was 1.72, 3.38, and 3.22 fold that of WF, and flavonoids content was 3.41, 10.53, and 10.66 times higher (all *p* < 0.01). These results highlight the formulations’ superiority in nutritional fortification and their potential to modulate blood glucose and lipid levels. Regarding differences among the formulations, the DF content of R3 was significantly greater (*p* < 0.01) than that of R1 and R2 (Figures 1a–g).

**Figure 1:**
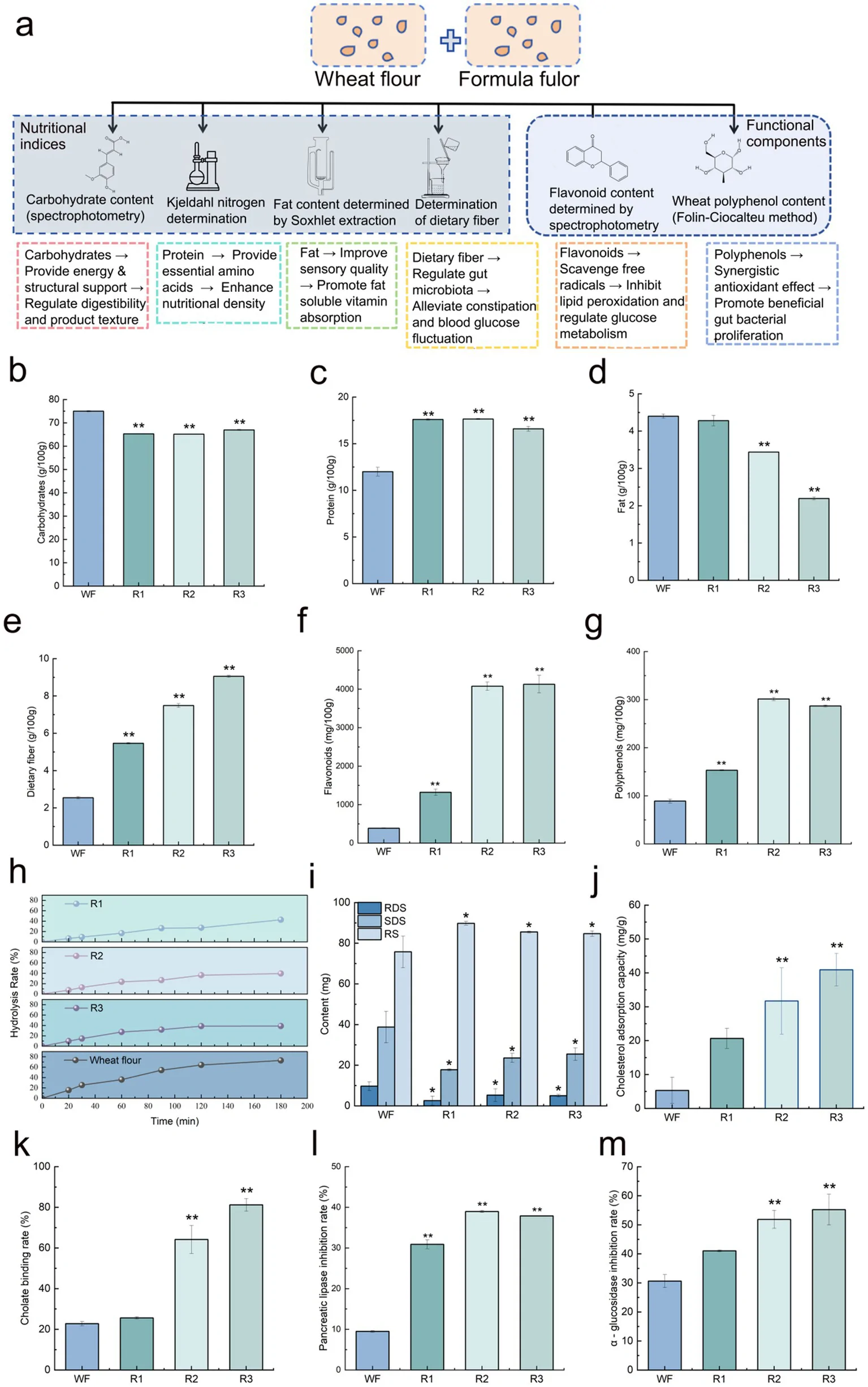
Nutritional and functional characterization of WF and formula powder, and their *in vitro* biological activities. **(a)** Nutritional composition and functional components (a). **(b)** Carbohydrate content. **(c)** Protein content. **(d)** Fat content. **(e)** DF content. **(f)** Flavonoid content. **(g)** Flavonoid content (FBG). **(h)** The hydrolysis rate. **(i)** Starch components. **(j)** Cholesterol adsorption capacity. **(k)** Cholate binding rate. **(l)** Pancreatic lipase inhibition ability. **(m)** α-Glucosidase inhibitory activity. *p < 0.05 and **p < 0.01 vs. WF, *n* = 3.

### 3.2 Starch hydrolysis rate and components

*In vitro* digestion analysis showed that all flour samples exhibited similar hydrolysis patterns, with the maximum hydrolysis rate occurring at 20 min followed by a gradual decline (Figure 1h). Analysis of starch fractions (Figure 2i) revealed that all formulated flours had significantly lower RDS and SDS contents, but significantly higher RS content, relative to WF (*p* < 0.05). WF exhibited a final hydrolysis rate (C∞) of 70.48 ± 1.33 and an eGI of 70.89 ± 0.42, in contrast, all three formulated flours showed significantly lower C∞ and eGI values (Table 2). Specifically, compared with WF, the C∞ values of R1, R2, and R3 decreased by 39.33, 44.08, and 46.82%, respectively, while their eGI values decreased by 45.77, 39.57, and 41.17%, respectively (Table 2).

**Figure 2:**
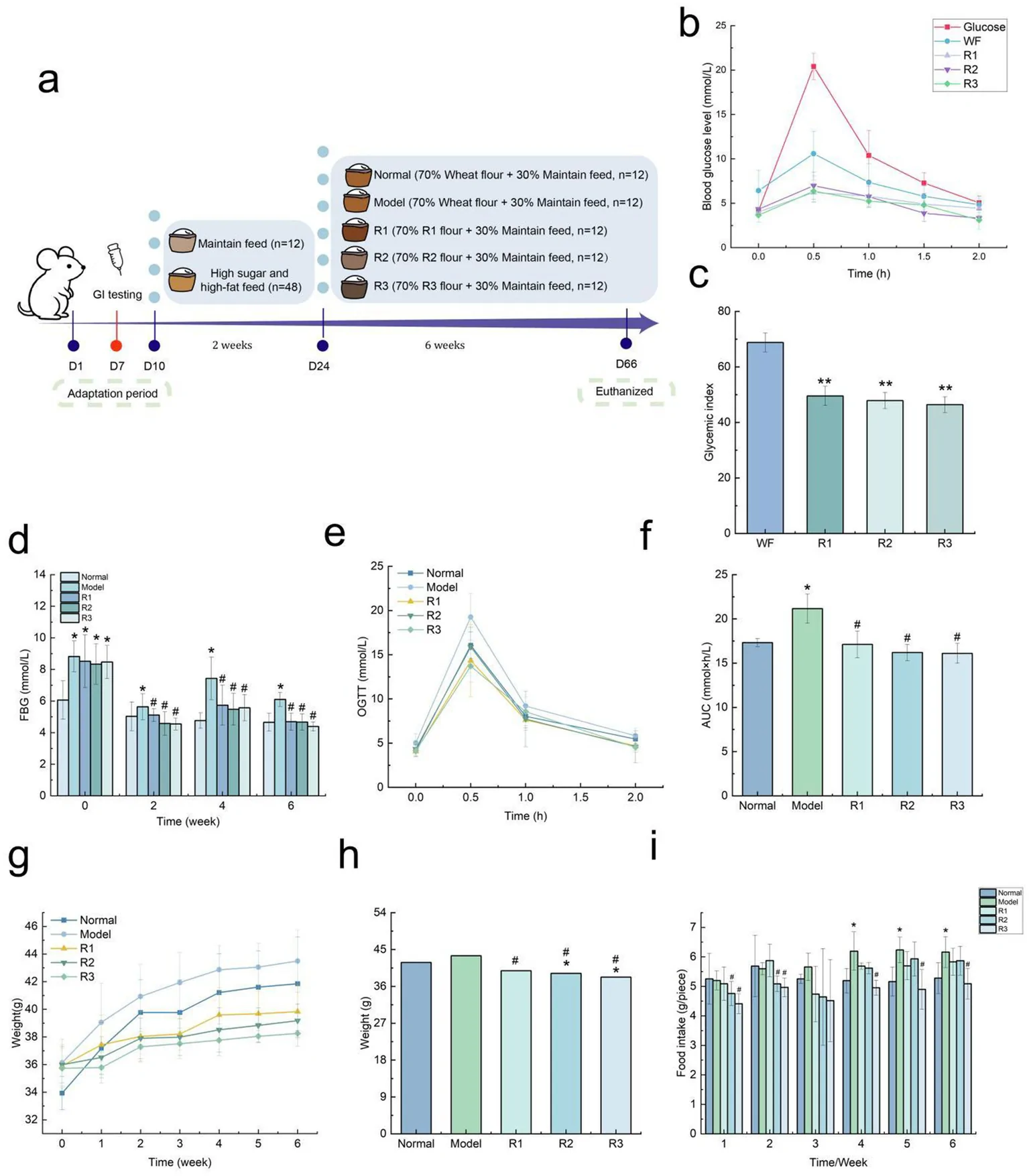
Effects of low-GI flours on glycemic control, body weight, and food intake in mice **(a)** Experimental design. **(b)** Blood glucose level **(c)** Glycemic index **(d)** α - glucosidase inhibitory activity (FBG) **(e)** Oral glucose tolerance test (OGTT) **(f)** Area under the curve (AUC) **(g)** Weekly weight changes **(h)** Final weight **(i)** Food intake. *p < 0.05 and **p < 0.01 vs. normal group; #*p* < 0.05 and ^##^*p* < 0.01 vs. model group, *n* = 6.

**Table 2:** *In vitro* digestion parameters of formulated flour.

ID	C∞	HI%	eGI
WF	70.48 ± 1.33	72.73 ± 0.49	70.89 ± 0.42
R1	42.76 ± 5.30^**^	35.08 ± 1.62^**^	38.44 ± 1.40^**^
R2	39.41 ± 2.99^**^	40.19 ± 0.79^**^	42.84 ± 0.68^**^
R3	37.48 ± 4.27^**^	38.87 ± 0.47^**^	41.70 ± 0.41^**^

Data are presented as mean ± SD (*n* = 3), C∞, final hydrolysis rate; HI, hydrolysis index; eGI, estimated glycemic index. *p < 0.05; **p < 0.01 compared with the WF.

### 3.3 *In vitro* lipid-lowering and hypoglycemic activity analysis

Cholesterol adsorption capacity is a critical indicator for evaluating the lipid-lowering potential of functional foods, and since cholesterol absorption primarily occurs in the small intestine (43), our experiments were conducted under simulated intestinal conditions (pH 7.00). The three composite flours-R1, R2, and R3 demonstrated significant cholesterol adsorption capacities of 20.65, 31.70, and 44.25 mg/g, respectively, showing highly significant differences (*p* < 0.01) compared to conventional WF (Figure 2j). Foods containing a large amount of DF can lower blood cholesterol levels, as enhanced bile salt binding promotes cholesterol catabolism, thereby reducing serum cholesterol levels (44). Formulations R2 and R3 exhibited significantly higher sodium cholate binding capacities (64.17 and 81.23%, respectively; *p* < 0.01) than WF. This enhanced activity may be attributed to their higher DF content, which is known to facilitate bile salt sequestration (Figures 1k, 2k).

The *in vitro* analyses demonstrated significant metabolic regulatory effects of the low-GI flours. Pancreatic lipase inhibition assays revealed that both WF and the three experimental low-GI flours exhibited inhibitory activity. However, the three formulas showed markedly enhanced efficacy (*p* < 0.05; Figures 1l, 2l). Regarding carbohydrate metabolism regulation, α-glucosidase inhibition assays yielded particularly noteworthy results. As α-glucosidase mediates the rate-limiting step of carbohydrate digestion by catalyzing disaccharide hydrolysis, its inhibition represents a validated strategy for postprandial glycemic control. The composite flours demonstrated highly significant α-glucosidase inhibitory activity compared to WF (*p* < 0.01), with R3 exhibiting the most potent effect (55.22% inhibition, representing a 22.22% enhancement over WF; (Figures 1m, 2m). These findings suggest that the low-GI flour exhibits favorable bioactive properties, including pronounced cholesterol adsorption capacity and pancreatic lipase inhibitory activity, indicating its potential to interfere with dietary lipid digestion. In addition, the α-glucosidase inhibitory activity of these formulations suggests a potential to modulate carbohydrate digestion, which may contribute to improved postprandial glycemic control. Collectively, these *in vitro* results support the potential of the tested formulations, particularly R3, as a dietary intervention for obesity-related metabolic disorders.

### 3.4 Effect of low-GI flour on postprandial glycemic response and glycemic index

The schematic diagram of the experimental protocol, including animal grouping, dietary interventions, and sampling time points, is presented in Figure 2a. On day 7 of the adaptation period. Following oral gavage, blood glucose levels in all groups reached their peak at 0.5 h and subsequently returned to baseline by 2 h. Notably, the three formula powder groups exhibited a slower postprandial glucose rise, lower peak values, and a more gradual decline compared with the glucose and WF groups (Figure 2b). Furthermore, the GI values of all three formula groups were significantly lower than that of the WF group (*p* < 0.01). These results indicate that the three flours possess glucose-lowering effects and can be classified as low-GI foods (Figure 2c).

### 3.5 Effect of low-GI flour on FBG

FBG is a primary diagnostic criterion for diabetes. In the normal group, FBG levels remained within the physiological range (3.9–6.1 mmol/L) throughout the experiment, whereas values exceeding 7.0 mmol/L were considered diabetic thresholds. At week 0, FBG levels in all high-fat diet groups (model, R1-R3) were significantly higher than those in the normal group (*p* < 0.05), indicating the successful induction of hyperglycemia. Over the 6-week intervention, all three treatment groups showed significant reductions in FBG relative to the model group (*p* < 0.05), with the R3 group exhibiting the greatest reduction at week 6. Pairwise comparisons showed that R3 was significantly lower than both R1 and R2 at week 6 (*p* < 0.05; Figure 2d). These results suggest that the low-GI flours may effectively ameliorate diet-induced hyperglycemia and serve as a potential dietary intervention for glucose management.

### 3.6 Effect of low-GI flour on glucose tolerance

The OGTT serves as a clinical diagnostic tool for evaluating glucose metabolism and insulin secretion capacity in diabetes diagnosis (45). As shown in Figure 3e, oral glucose administration induced a rapid glycemic excursion in all groups, with maximum concentrations observed at 0.5 h, followed by a progressive return toward baseline over the subsequent 1.5 h. Throughout the testing period, the model group maintained significantly higher blood glucose levels than the other groups at all time points (Figure 2e; *p* < 0.05). The AUC analysis provided quantitative assessment of glucose tolerance. The model group displayed substantially higher AUC values relative to both control and treatment groups (*p* < 0.01), indicating impaired glucose tolerance. In contrast, all three intervention groups exhibited significantly lower AUC values compared to the model group (*p* < 0.01; Figure 2f). These findings suggest that low-GI flour formulations, particularly in the R3 group, which was associated with improved glucose tolerance and enhanced glycemic control in obese mice.

**Figure 3:**
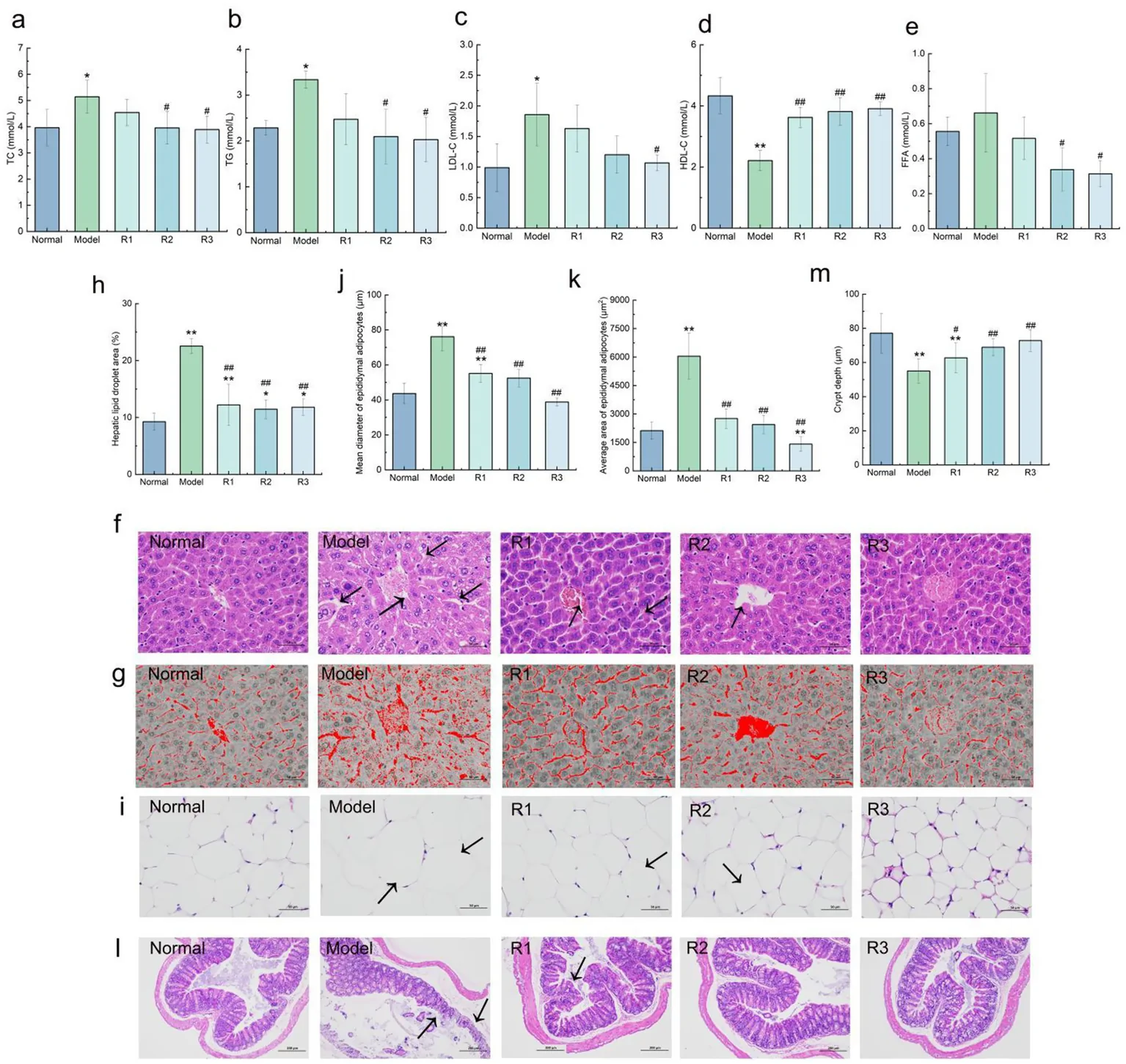
Low-GI regulates lipid metabolism and tissue histopathology in mice. **(a)** Total cholesterol. **(b)** Triglyceride. **(c)** Low-density lipoprotein cholesterol. **(d)** High-density lipoprotein cholesterol. **(e)** Free fatty acid. **(f)** Histopathology of the liver. **(g)** ImageJ-based pseudo-coloring of hepatic vacuoles in H&E-stained sections. **(h)** ImageJ-based quantification of hepatic vacuolation. **(i)** Adipose tissue histopathology. **(j)** Quantification of epididymal adipocyte diameter. **(k)** Quantification of epididymal adipocyte area. **(l)** Histopathology of the cecal tissue. **(m)** Quantification of cecal crypt depth. *p < 0.05; **p < 0.01, compared to the normal group; *^#^p < 0.05*; *^##^p* < 0.01, compared to the model group, a-e:n = 6, f-m:n = 3.

### 3.7 Body weight

Body weight changes serve as a crucial indicator for directly assessing obesity progression in mice. As illustrated in Figure 2g, following 2 weeks of high-fat diet induction, a significant divergence in body weight emerged between the normal group and the experimental groups (*p* < 0.05), confirming the successful establishment of the obesity model. Throughout the 6-week intervention period, the normal and model groups reached final body weights of 41.85 ± 3.37 g and 43.50 ± 2.26 g, respectively. Among the intervention groups, the R1 group was the highest mean weight (39.84 ± 1.95 g), followed by R2 (39.18 ± 1.19 g), while the R3 group maintained the lowest final weight (38.25 ± 0.92 g; Figure 2h). As shown in Table 3, liver and kidney indices remained unchanged across all groups (*p* > 0.05), indicating no hepatotoxicity or nephrotoxicity. In contrast, the R3 group exhibited significantly lower epididymal and perirenal fat coefficients (1.43 ± 0.37% and 0.47 ± 0.14%, respectively) and Lee’s index (7.22 ± 0.13) compared with the model group (3.10 ± 0.68%, 1.05 ± 0.16%, and 7.53 ± 0.11, respectively; *p* < 0.05). Spleen and cardiac indices in the R1, R2, and R3 groups were also significantly lower than in the model group (*p* < 0.05), returning to levels comparable to those of the normal control group, which may reflect alleviation of obesity-related splenic and cardiac alterations (46, 47). These data indicate that R3 feeding was associated with reduced visceral fat accumulation and improvement in obesity-related organ parameters without adverse effects on major metabolic organs.

**Table 3:** Organ indices of mice in each group.

Index (%)	Normal	Model	R1	R2	R3
Liver index	4.10 ± 0.16	4.46 ± 0.32*	3.91 ± 0.45	3.99 ± 0.30	3.96 ± 0.20
Kidney index	1.59 ± 0.12	1.69 ± 0.14	1.59 ± 0.13	1.58 ± 0.09	1.54 ± 0.06
Spleen index	0.28 ± 0.03	0.33 ± 0.04	0.28 ± 0.028^#^	0.28 ± 0.04^#^	0.28 ± 0.03^#^
Cardiac index	0.61 ± 0.10	0.67 ± 0.10	0.60 ± 0.10	0.59 ± 0.09	0.58 ± 0.06^#^
Epididymal fat coefficient	2.81 ± 0.49	3.10 ± 0.68	2.49 ± 0.48^#^	2.38 ± 0.65^#^	1.43 ± 0.37^#^
Perirenal fat coefficient	0.89 ± 0.22	1.05 ± 0.16*	0.77 ± 0.22^#^	0.76 ± 0.22^#^	0.47 ± 0.14^#^
Lee’s Index	7.36 ± 0.19	7.53 ± 0.11*	7.37 ± 0.123^#^	7.33 ± 0.09^#^	7.22 ± 0.13^#^

Data are presented as mean ± SD (*n* = 12), *p < 0.05 vs. normal group; #*p* < 0.05 vs. model group.

### 3.8 Food intake and energy utilization

To determine whether the reduced weight gain in R3-fed mice was associated with decreased food intake, we calculated weekly food intake, energy intake, and feed efficiency (Figure 3i; Table 4). Over the 6-week intervention period, the average daily food intake was lowest in the R3 group (4.81 ± 0.27 g/day) and highest in the model group (5.84 ± 0.42 g/day), with the normal, R1, and R2 groups showing intermediate values (5.31 ± 0.21, 5.49 ± 0.46, and 5.32 ± 0.57 g/day, respectively). The energy densities of the complete diets, calculated using the Atwater factors based on dietary composition (70% flour + 30% basal diet), were 3.77 kcal/g for normal and model groups, 3.70 kcal/g for R1, 3.66 kcal/g for R2, and 3.63 kcal/g for R3. Total daily energy intake per mouse was lowest in the R3 group (17.44 ± 1.00 kcal/day), representing a 20.8% reduction compared with the model group (22.02 ± 1.59 kcal/day). Feed efficiency, calculated as body weight gain per gram of food consumed, was substantially lower in the R3 group (0.013 g/g) than in the model group (0.030 g/g). These data indicate that R3 flour reduced food intake and feed efficiency in obese mice. The decrease in food intake may be attributable to the higher dietary fiber content of R3 flour, which promotes satiety (48).

**Table 4:** Intake food, energy intake, and feed efficiency.

Groups	Intake food (g/mouse/day)	Energy density (kcal/g)	Total energy intake (kcal/mouse/day)	Weight gain (g)	Feed efficiency (g/g)
Normal	5.31 ± 0.21	3.77	20.00 ± 0.73	7.30 ± 1.56	0.033
Model	5.84 ± 0.42*	3.77	22.02 ± 1.59*	7.34 ± 1.30	0.030
R1	5.49 ± 0.46	3.70	20.30 ± 1.72	4.09 ± 0.97^#^	0.018
R2	5.32 ± 0.57^#^	3.66	19.47 ± 2.07^#^	3.22 ± 1.12^#^	0.014
R3	4.81 ± 0.27^##^	3.63	17.44 ± 1.00^##^	2.54 ± 1.34^#^	0.013

*p < 0.05; **p < 0.01 vs. normal group, #*p* < 0.05, ##*p* < 0.01 vs. model group.

### 3.9 Effects of low-GI flour on serum lipid parameters

The 6-week dietary intervention with low-GI lipid-lowering flour significantly improved serum lipid profiles in experimental mice. Compared to the model group, all treatment groups exhibited reduced serum TC and TG levels (*p* < 0.05). The R3 group demonstrated the most pronounced improvements, with serum TC, TG, and LDL-C levels decreasing by 24.34, 39.18, and 42.54%, respectively (Figures 3a–c; *p* < 0.05) relative to model group. Furthermore, HDL-C levels were significantly elevated in all treatment groups (R1-R3) following dietary regulation with the low-GI flour (*p* < 0.01; Figure 3d). Serum FFA levels were also significantly reduced in the R2 and R3 groups compared with the model group, with decreases of 49.00 and 52.69%, respectively (*p* < 0.05; Figure 3e), among them, the R3 group showed the greatest reduction. These results indicate that chronic consumption of low-GI functional flour may contribute to the amelioration of diet-induced dyslipidemia by modulating key serum lipid parameters.

### 3.10 Morphological analysis of liver tissue

Histological analysis revealed that a high-sugar and high-fat diet induces significant alterations in hepatic morphology, including cytoplasmic vacuolization of hepatocytes, and neutrophil infiltration, ultimately leading to hepatic steatosis and liver injury. The liver tissue of mice in the normal group displayed normal histological architecture with uniformly distributed hepatocytes and absence of cellular division. In contrast, the model group exhibited disrupted hepatic morphology, characterized by disorganized hepatocyte arrangement and substantial accumulation of lipid droplets (Figure 3f). Dietary intervention with the three low-GI flours effectively ameliorated pre-existing diet-induced hepatic injury in obese mice, as evidenced by restored hepatocyte arrangement, preserved tissue integrity, and reduced cytoplasmic vacuolation. Quantitative morphometric analysis using ImageJ further revealed that the percentage of lipid droplet area relative to the total field area was significantly lower in the low-GI flour groups than in the model group (*p* < 0.01; Figures 3g,h). Collectively, these findings indicate that low-GI flours effectively alleviate hepatic lipid accumulation established after high-fat diet feeding.

### 3.11 Morphological analysis of epididymal adipose tissue

The size of white adipocytes serves as a key indicator of systemic fat deposition, with epididymal fat representing a major subtype. Histopathological examination of adipose tissue sections (Figure 3i) revealed that epididymal adipocytes in the normal group exhibited uniform size, regular morphology, and well-preserved tissue structure. In contrast, the model group displayed marked adipose tissue dysfunction, characterized by disorganized adipocyte arrangement, cellular hypertrophy, and significant size heterogeneity. We used ImageJ to quantitatively assess adipocyte cross-sectional area and radius, further revealing that adipocyte enlargement is directly associated with obesity progression. Statistical analysis showed that, compared with the model group, dietary interventions with R1, R2, and R3 significantly reduced adipocyte radius and cross-sectional area (*p* < 0.01; Figures 3j,k).

### 3.12 Morphological analysis of cecal tissue

Histopathological evaluation of cecal sections revealed distinct morphological differences between experimental groups (Figure 3l). The normal group displayed intact cecal architecture, characterized by densely arranged crypts of uniform length, normal intestinal wall thickness, and absence of mucosal damage. In stark contrast, the model group exhibited severe mucosal injury, manifesting as irregular crypt arrangement, structural deformities, and crypt dropout. Dietary intervention with R1, R2, and R3 markedly improved these pathological changes, with cecal morphology approaching that of the normal group and showing reduced mucosal epithelial damage. Quantitative morphometric analysis using ImageJ further revealed that cecal crypt depth was significantly reduced in the model group compared to the normal group (*p* < 0.01). However, treatment with R1, R2, and R3 significantly restored crypt depth compared to the model group (*p* < 0.05, *p* < 0.01, and *p* < 0.01, respectively; Figure 3m). These results indicate that the composite flour formulations effectively ameliorate diet-induced structural alterations in cecal tissue.

### 3.13 Microbial community analysis of cecal contents

As the primary site of microbial fermentation in mice, cecal microbiota composition was characterized through 16S rRNA sequencing analysis. Out analysis revealed distinct microbial profiles among groups (Figure 4a), with the model, normal, and R3 groups containing 401, 446, and 491 unique OTUs respectively, demonstrating significant intergroup variation in microbial community structure. Especially, suggesting an association between R3 intervention and shifts in microbial composition toward a phenotype more similar to that of normal mice.

**Figure 4:**
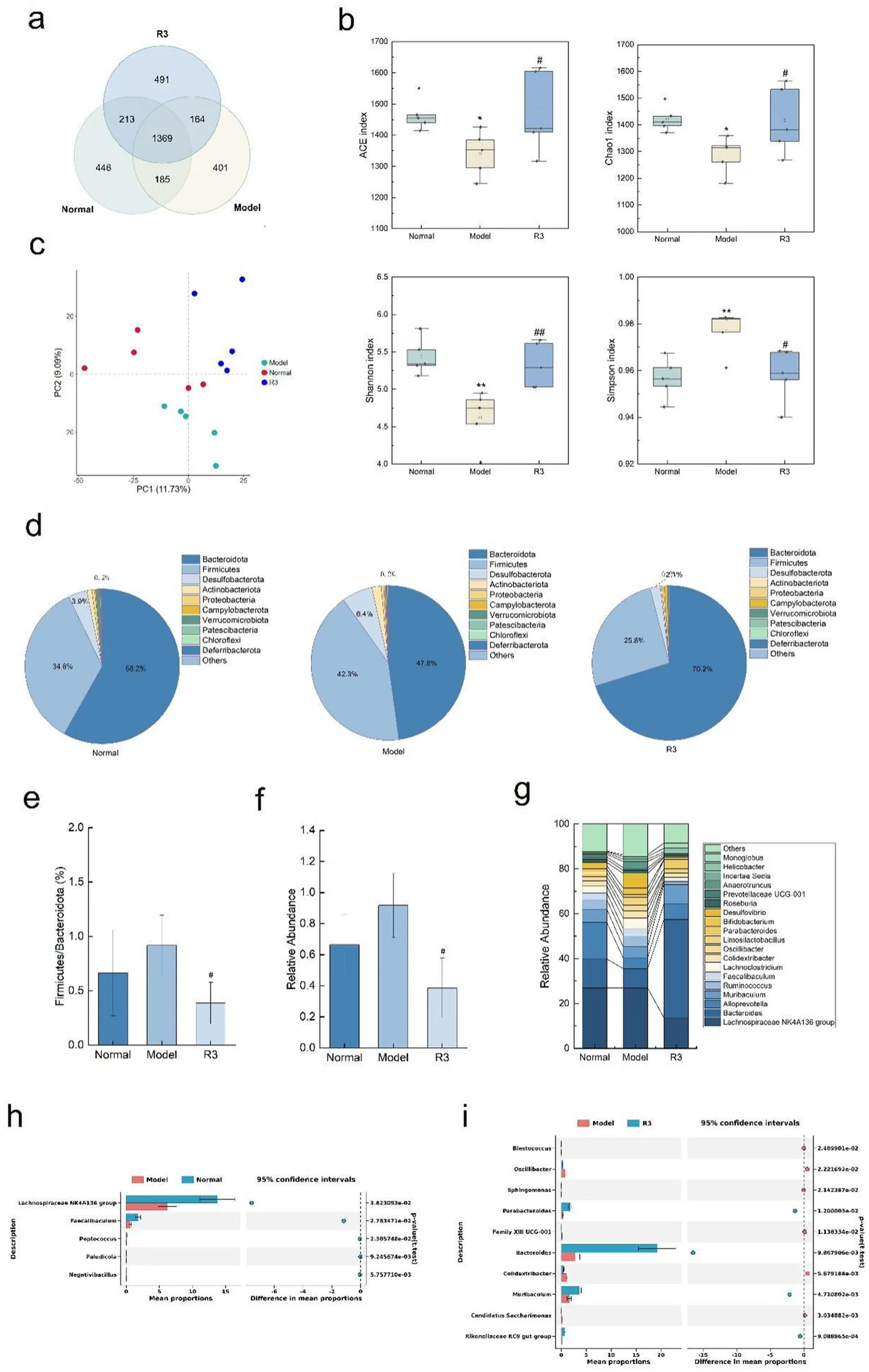
Compositions and differences in gut microbiota. **(a)** Venn diagram of the gut microbiota in mice. **(b)** Analysis of α-diversity within the groups: ACE index, Chao1 index, Shannon’s index and Simpson’s index. **(c)** PCoA. **(d–f)** Relative abundance of the intestinal microbiota community at the phylum, and genus levels. **(g)** Changes in the relative abundance of intestinal microbiota at the genus level in each group of mice. **(h,i)** Bar chart of intestinal microbiota expansion error in each group. *p < 0.05, **p < 0.01 compared to the normal group; *^#^p* < 0.05, *^##^p* < 0.01 compared to the model group, *n* = 5.

Comprehensive diversity analysis revealed significant differences in microbial ecology among groups. Species richness metrics (Chao1 and ACE indices) and diversity indicators (Shannon and Simpson indices) revealed marked differences between groups (Figure 4b). Compared to the normal group, the model group showed significantly reduced Chao1, ACE, and Shannon indices along with elevated Simpson index values (*p* < 0.05), indicating high-fat diet-induced impairment of microbial diversity. Importantly, the R3 intervention substantially reversed these effects, showing significant increases in ACE (*p* < 0.05), Chao1 (*p* < 0.05), and Shannon (*p* < 0.01) indices relative to the model group, while reducing Simpson index values (*p* < 0.01).

PERMANOVA based on Bray-Curtis distances revealed significant differences in microbial community structure among groups (*F* = 4.52, *R*^2^ = 0.31, *p* = 0.018). Principal component analysis revealed distinct clustering patterns, with the R3 group microbiota showing greater similarity to the normal group than to the model group (Figure 4c). This structural reorganization suggests that the low-GI flour intervention was associated with modulation of diet-induced dysbiosis, as microbial community composition shifted patterns more similar to those of the normal group. These findings suggest that targeted dietary interventions are associated with alterations in gut microbial ecology, potentially contributing to their metabolic benefits.

### 3.14 Low-GI flour regulates the intestinal contents at different categorical levels

A 6-week dietary intervention using low-GI flour significantly modulated the gut microbiota composition at both the phylum and genus levels (Figure 4d). The murine gut microbiota was predominantly composed of 5 major phyla: Bacteroidetes, Firmicutes, Desulfobacterota, Actinobacterota, and Proteobacterota, collectively representing >90% of the total microbial community. Compared with the normal group, high-fat diet feeding induced prominent phylum-level microbial alterations, including a 22.13% significant increase in Firmicutes abundance and a concomitant 17.82% reduction in Bacteroidetes abundance, resulting in a 0.253 elevation in the F/B ratio. Notably, the F/B ratio serves as a well-established biomarker for assessing gut dysbiosis (49). In contrast, the R3 intervention group effectively reversed such microbial disorders: relative to the model group, this group presented a 39.09% decrease in Firmicutes and a 46.73% increase in Bacteroidetes, with the F/B ratio markedly reduced by 0.53 (*p* < 0.05; Figure 4e). This reduction suggests that the low-GI flour was associated with normalization of the F/B ratio in obese mice. These results indicate that the low-GI flour was associated with normalization of the F/B ratio in obese mice, suggesting that dietary modification with functional low-GI flour may contribute to the modulation of obesogenic diet-induced microbial dysbiosis.

At the genus level, significant differences (*p* < 0.05) were observed among the three groups of mice, with *Lachnospiraceae* NK4A136 group, *Bacteroides*, *Alloprevotella*, *Muribaculum*, *Ruminococcus*, and *Faecalibaculum* emerging as the most dominant bacterial taxa across all groups (Figures 4f,g). Following 6 weeks of dietary intervention, substantial alterations in gut microbiota composition were detected in all three groups. Compared to the normal group, the model group exhibited significant decreases in *Lachnospiraceae NK4A136 group, Faecalibaculum、Peptococcus, Paludicola* and *Negativibacillus* (Figure 4h). In contrast, the R3 group demonstrated a marked increase in the *Bacteroides, Muribaculum, Parabacteroides* and *Rikenellaceae rc9 gut group*, while the abundances of *Blastococcus*, *Sphingomonas*, *Candidatus saccharimonas, Oscillibacter, Family XII UCG-001, Colidextribacter* were significantly reduced compared to the model group (Figure 4i). Previous studies have reported that individuals with high-fat diets exhibit lower relative abundances of *Bacteroides* and *Faecalibacterium*, whereas increases in *Bacteroidetes* and *Parabacteroides* have been documented (50). These findings suggest that low-GI flour is associated with altered relative abundance of obesity-associated bacteria at the genus level, which may correlate with improvements in obesity-related metabolic parameters.

### 3.15 Analysis of SCFAs content in cecal contents of mice

SCFAs are key metabolites produced by bacterial fermentation of DF in the gastrointestinal tract, serving as important energy sources for intestinal epithelial cells (51). RS has been also reported to enhance SCFA production and modulate gut microbial metabolism (52). In the present study, quantitative analysis of cecal SCFAs revealed that the model group showed no significant differences in acetic, propionic, butyric, isobutyric, valeric, and isovaleric acid contents compared with the normal group (*p* > 0.05), potentially due to the limited nutritional components provided by the WF diet. In contrast, the R3 intervention group exhibited significant increases in acetic, propionic, butyric, and isobutyric acid contents compared with the model group, with increases of 66.97, 71.00, 66.89, and 182.15%, respectively (*p* < 0.05). Meanwhile isovaleric acid content was significantly decreased by 59.23% (*p* < 0.05) in the R3 group compared with the model group, whereas valeric acid content showed an increasing trend without statistical significance (Figures 5a–f). Related studies have shown that RS2 increased the content of butyric acid in the colon and reduced the levels of isobutyric acid and isovaleric acid. In this study, R3 flour intervention increased the content of isobutyric acid and reduced the content of isovaleric acid. This may be due to the complex composition of R3 flour, which differs from pure RS2 supplementation (53).

**Figure 5:**
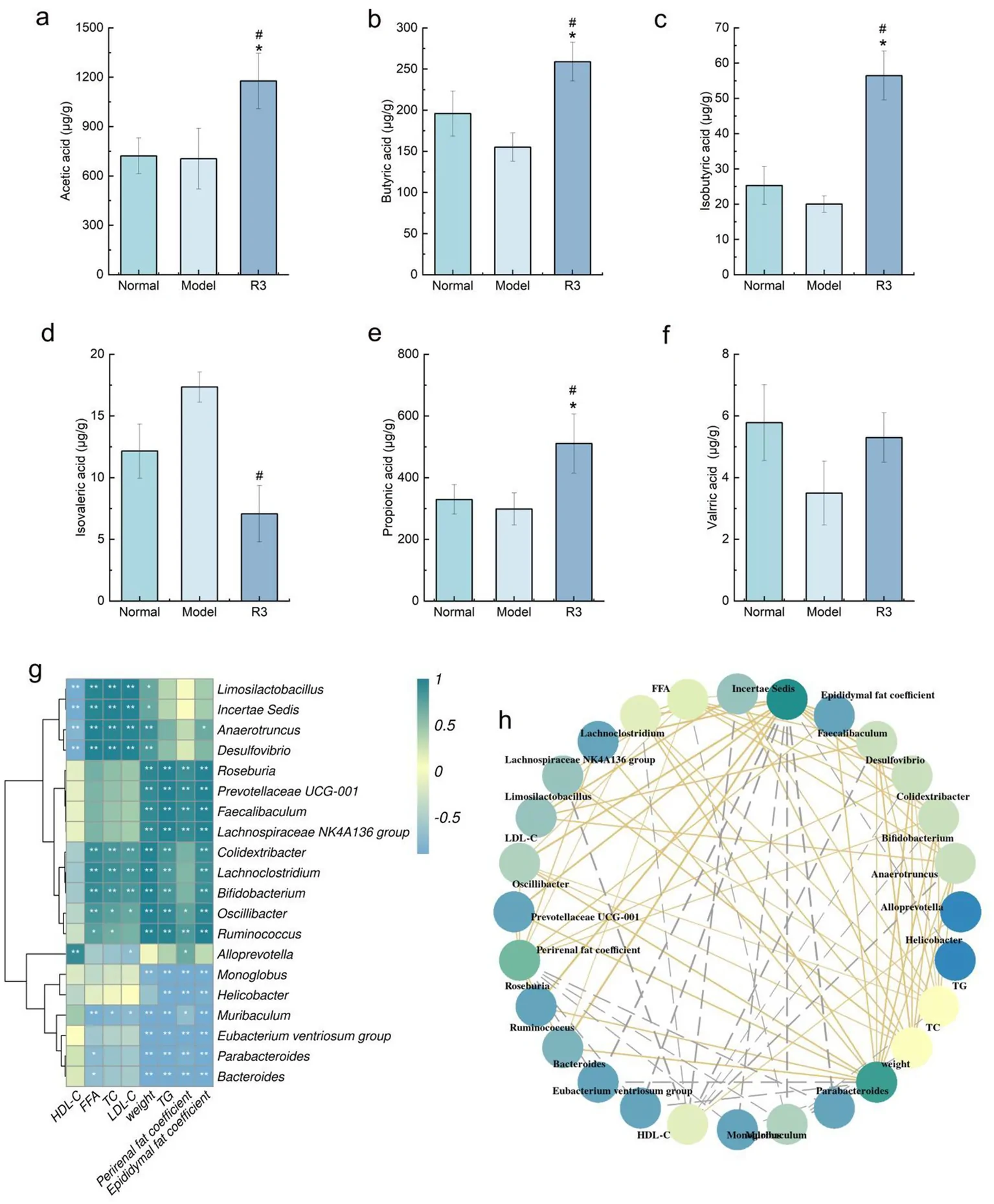
Effects of R3 on SCFAs and organic acids in feces and results of Spearman’s correlation analysis. **(a)** Acetic acid. **(b)** Propionic acid. **(c)** Butyric acid. **(d)** Valeric acid. **(e)** Isobutyric acid. **(f)** Isovaleric acid. **(g,h)** Correlation analysis results. *p < 0.05, compared to the normal group; *^#^p* < 0.05, compared to the model group, *n* = 5.

The upregulated SCFA levels were significantly correlation with the observed restructuring of gut microbiota, particularly changes in taxa related to SCFAs production, such as the reduced F/B ratio (54). Enhanced SCFA production has been associated with gut barrier protection and immune modulation toward a tolerogenic, anti-inflammatory state. These findings suggest that the low-GI flour, particularly the R3 formulation, was associated with enhanced microbial metabolite production in obese mice.

### 3.16 Correlational analysis

To explore the relationships among serum biochemical indices, body weight, and gut microbiota, we constructed correlation heatmaps and network diagrams based on the top 20 abundant genera (Figures 5g,h). The results revealed complex and genus-specific correlation patterns. *Anaerotruncus*, *Limosilactobacillus*, *Incertae Sedis*, and *Desulfovibrio* exhibited positive correlations with LDL-C, TC, and FFA, while showing negative correlations with HDL-C. *Roseburia*, *Prevotellaceae* UCG-001, *Faecalibaculum*, and *Lachnospiraceae* NK4A136 group were positively associated with body weight, TG, epididymal fat index, and perirenal fat index. *Colidextribacter*, *Lachnoclostridium*, and *Bifidobacterium* showed positive correlations with FFA, TC, LDL-C, body weight, TG, and epididymal fat index. Although *Bifidobacterium* has been widely recognized as a beneficial probiotic, capable of improving glucose homeostasis, inhibiting weight gain, and preventing fat accumulation (55), the metabolic effects of *Bifidobacterium* are strain specific, and different strains even exhibit different or even opposite effects on host metabolism (56). For instance, a study in obese children reported that *Bifidobacterium longum* subsp. infantis and *Bifidobacterium breve* are higher in obese infants, and the relative abundance of *Bifidobacterium bifidum* and *Bifidobacterium longum* subsp. longum decreases (57). This suggests that the genus-level positive correlation observed in our study may be driven by the expansion of specific subspecies under obese conditions, while other subspecies with beneficial effects are reduced.

*Oscillibacter* and *Ruminococcus* were positively correlated with body weight, TG, and epididymal fat index. In addition, *Monoglobus*, *Eubacterium ventriosum* group, *Parabacteroides*, and *Bacteroides* showed negative correlations with body weight, TG, epididymal fat index, and perirenal fat index. *Helicobacter* was negatively correlated with TC, epididymal fat index, and perirenal fat index. Additionally, *Muribaculum* has been linked to elevated propionic acid levels and modulation of the CYP7A1/FXR/SREBP-2/LDLR signaling pathway (58). In this study, *Muribaculum* was negatively correlated with epididymal fat index, TG, body weight, and FFA. These correlational analyses reveal complex and significant associations between gut microbial genera and host metabolic indices, particularly involving genera such as *Muribaculum, Bacteroides*, and *Bifidobacterium*. However, as these are correlational findings, they do not establish causality and require further functional validation.

## 4 Conclusion

In recent years, significant progress has been made in the field of low-GI foods. Using the rich bioactive components of selected raw materials, including *L. japonica*, *L. edodes*, tartary buckwheat, kudzu root, and konjac, we formulated a low-GI blended flour and evaluated its functional properties. This study indicates that these low-GI flours exhibit higher dietary fiber content and enhanced biological activities. This blended flour, particularly the R3 formulation, demonstrated the highest sodium cholate and cholesterol adsorption capacities, potent inhibitory effects on pancreatic lipase and α-glucosidase, as well as hypoglycemic and lipid-lowering properties *in vitro*. In addition, 16S rRNA sequencing revealed that R3 intervention was associated with favorable shifts in gut microbial composition, including increased microbial diversity (ACE, Chao1, Shannon), a reduced F/B ratio, and enriched abundances of beneficial genera including *Bacteroides*, *Muribaculum*, and *Parabacteroides*. Collectively, these findings suggest that this low-GI functional flour, especially the R3 formulation, may serve as a dietary intervention for improving glycemic and lipid metabolism.

## 5 Study limitations

This study has some limitations that warrant further investigation. First, the functional flour is a complex mixture of dietary fibers, polyphenols, and other bioactive constituents; the specific components responsible for the observed anti-obesity and microbiota-modulating effects remain to be identified. Second, although our correlation analyses revealed significant associations between gut microbial shifts and improved metabolic outcomes, causal inference was not established. Future studies employing fecal microbiota transplantation into germ-free recipients or antibiotic-treated mice are needed to determine whether the gut microbiota directly mediates these effects and to elucidate the downstream host signaling cascades. Third, the host signaling pathways downstream of SCFA production, such as SCFA-sensing G-protein-coupled receptors (e.g., GPR41/GPR43) and their effects on gut hormone secretion, were not examined in the present study. Additionally, the reduced feed efficiency observed in R3-fed mice suggests a potential alteration in energy metabolism; however, direct evidence of changes in energy. Expenditure- or substrate oxidation would require indirect calorimetry, which was not performed in this study. Addressing these questions in future studies will help elucidate the mechanisms underlying the metabolic benefits of this low-glycemic index functional flour.

## Data Availability

The original contributions presented in the study are publicly available. This data can be found in the NCBI repository: https://www.ncbi.nlm.nih.gov/bioproject/PRJNA1525171/.
